# Bio-inspired spatially variant photonic crystals for self-collimation and beam-steering applications in the near-infrared spectrum

**DOI:** 10.1038/s41598-021-97608-6

**Published:** 2021-09-21

**Authors:** Rudra Gnawali, Andrew Volk, Imad Agha, Tamara E. Payne, Amit Rai, Jimmy Touma

**Affiliations:** 1 Applied Optimization, Inc., 3040 Presidential Dr. Suite 100, Fairborn, OH 45324 USA; 2 Department of Electro-Optics and Photonics, University of Dayton, Dayton, OH 45469 USA; 3 Department of Physics, University of Dayton, Dayton, OH 45469 USA; 4 Air Force Research Laboratory, Munitions Directorate, Eglin Air Force Base, FL 32542-6810 USA

**Keywords:** Applied optics, Optical materials and structures, Optical techniques

## Abstract

The self-collimation of light through Photonic Crystals (PCs) due to their optical properties and through a special geometric structure offers a new form of beam steering with highly optical control capabilities for a range of different applications. The objective of this work is to understand self-collimation and bending of light beams through bio-inspired Spatially Variant Photonic Crystals (SVPCs) made from dielectric materials such as silicon dioxide and common polymers used in three-dimensional printing like SU-8. Based upon natural PCs found in animals such as butterflies and fish, the PCs developed in this work can be used to manipulate different wavelengths of light for optical communications, multiplexing, and beam-tuning devices for light detection and ranging applications. In this paper, we show the optical properties and potential applications of two different SVPC designs that can control light through a 90-degree bend and optical logic gates. These two-dimensional SVPC designs were optimized for operation in the near-infrared range of approximately 800–1000 nm for the 90-degree bend and 700–1000 nm for the optical logic gate. These SVPCs were shown to provide high transmission through desired regions with low reflection and absorption of light to prove the potential benefits of these structures for future optical systems.

## Introduction

Photonic Crystals (PCs) are periodic nanostructures designed to affect the motion of photons in the same way that the periodic potential in a semiconductor crystal affects the electron motion by defining allowed and forbidden electronic energy bands^1^. Spatially Variant Photonic Crystals (SVPCs) have been studied in the past and designed using materials that have a low-refractive index; they have shown their capability to adiabatically control light beams with high polarization selectivity^2,3^. Two different polarizations can be considered when looking at the optical applications and functions of different devices such as PCs. In p-polarization (Transverse Magnetic–TM), the electric field is parallel to the plane of incidence, while in s-polarization (Transverse Electric–TE), the electric field is perpendicular to the plane of incidence. Optical devices can be designed and tailored to manipulate these different polarizations of light through their geometries and properties such as the refractive index and extinction coefficient. PCs and metamaterials exhibit dispersion properties that have been used for super prisms, negative refraction, and dispersion compensation^1,4^. In order to bend a beam of light, Spatially Variant Lattices (SVL) can be created by decomposing the lattice into a set of planar gratings. Each of these planar gratings are spatially varied individually. As a result, the entire geometry has a uniform variance^1,5,6^, and the unit cell of the structure is expanded into a Fourier series along its reciprocal lattice vectors.

**Figure 1:**
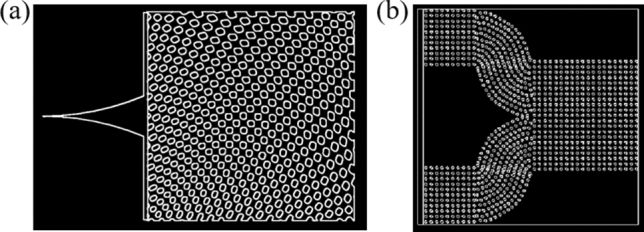
2D SVPC models for (**a**) scaled 90-degree bend and (**b**) simplified optical logic gate, i.e., OR gate. Models were developed through a combination of MATLAB and Computer Aided Design (CAD) software in SolidWorks and Lumerical.

There are many different classifications and types of PCs. Each PC has different advantages and disadvantages over the others in their ease of fabrication and applications. In order to create a structure that would allow us to finely tune the collimation and transmission of light in the near-infrared (IR) wavelength, a target wavelength for free-space optical applications, a PC based on an SVL was selected. This structure allows for self-collimation of a light beam through the structure by a bandgap control mechanism, preventing the need for a waveguide when bending light since all bending of light is a result of the SVL itself. The SVL design took inspiration from PC structures found in nature, most notably in butterfly wings. Animals such as butterflies and fish have evolved with gyroid or lattice structure PCs on their wings or scales that give them unique colors through their manipulation of light. We designed and simulated two-dimensional (2D) PCs in the near-IR wavelength using Finite Difference Time Domain (FDTD) simulations for two different design applications with a range of low-refractive index materials. The design for controlling light through a 90-degree bend is shown in Fig. 1a, and the design for an OR logic gate is shown in Fig. 1b. In order to give a better view of the structure, these models have been scaled to a smaller number of unit cells than those used in simulations.

The transmission, reflection, and absorption results from the simulation of these structures showed the optimal operational wavelengths for these designs as well as their merit for each application. While SVPCs have been shown to be capable of self-collimation and beam-steering applications in visible and microwave wavelengths^1,7^, this work is unique in that it has been applied to the near-IR range, allowing for new applications. Previously, the team had shown the ability of SVPCs composed of Indium Tin Oxide (ITO) to control light through a 90-degree bend^2^. These ideas are now expanded to additional materials and applications. Applying the properties and functions discussed in this paper to further applications such as optical logic gates or multiplexers and demultiplexers is also a new innovation that can be improved upon and implemented in future work. Systems such as LIght Detection And Ranging (LIDAR), optical communication systems, data transfer systems, and many more optical and electrical systems could use these SVPC structures for improved performance. Current logic gates work through electrical systems, but applying PCs to serve as logic gates in the near-IR range is a unique innovation that could greatly improve the speed and capacity of logic gates in the future.

## Methods

### Development of PCs and spatial variation

After investigating different geometric structures of SVPCs, the model based on a varying lattice proved to be the ideal structure for our applications due to its ability to be fabricated with a quality design and tuned for different frequencies. Similar structures can be found in nature such as the wings of some butterflies and scales of fish that enable unique optical characteristics that manipulate light. In this work, a combination of MATLAB and SolidWorks was used to generate the structures based on various equations for lattice structures and lattice constants for the selected materials. MATLAB was first used to generate Standard Tessellation Language (STL) files for geometric structures such as unit cells and overall lattices based on material parameters and spatial variation. These geometries from MATLAB were then imported into SolidWorks where individual layers could be stacked, simplified, or scaled to fit the desired parameters that could be used in Lumerical for FDTD simulations. The following equations were used to generate the unit cell parameters and accompanying lattice structures:

1 $$\overrightarrow{K}={K}_{x}\widehat{X}+{K}_{y}\widehat{Y}+{K}_{z}\widehat{Z};{K}_{x}=\frac{2\pi p}{{\Lambda }_{x}};{K}_{y}=\frac{2\pi q}{{\Lambda }_{y}};{K}_{z}=\frac{2\pi r}{{\Lambda }_{z}}$$

where $\overrightarrow{K}$ is the grating vector of the lattice; ${\overrightarrow{{K}_{x}}, \overrightarrow{{K}_{y}}, \overrightarrow{{K}_{z}}}$ are the grating vectors for $x, y,$ and $z$ directions; *p, q,* and *r* are integers; and ${\Lambda }_{x},{\Lambda }_{y}$ and ${\Lambda }_{z}$ are the grating periods for each direction^1^. The lattice constant determines the fill fraction of the SVL. The fill fraction is given by Eq. (2) as follows:

2 $$Ff=\frac{\frac{4}{3}\pi ({{d}_{{e}_{1}}}^{3}({t}_{{e}_{1}})+{{d}_{{e}_{2}}}^{3}({t}_{{e}_{2}}))}{a}$$

where subscripts ${e}_{1}$ and ${e}_{2}$ represent the first and second elements in the compound, respectively; *Ff* is the fill fraction; the value for *a* for each element is the lattice constant; the value for *t* is the number of atoms in a molecule; and *d* is the atomic radius. To generate the model for simulation, ${d}_{{e}_{1}}$ and ${d}_{{e}_{2}}$ were the atomic radii of the two elements in our models.

In order to control the bending of light, spatial variation must be introduced into the lattice; this can be achieved by decomposing the lattice into a set of planar gratings that are individually spatially varied, allowing the entire geometry to have uniform variance^1^. The unit cell of the structure is expanded into a Fourier series along its reciprocal lattice vectors $\overrightarrow{{T}_{1}}, \overrightarrow{{T}_{2}},\overrightarrow{{T}_{3}}$ that can be expressed as:

3 $$n\left(\overrightarrow{r}\right)={\sum }_{p,q,r}{a}_{p,q,r}{e}^{j\left(p\overrightarrow{{T}_{1}}+q \overrightarrow{{T}_{2}}+r\overrightarrow{{T}_{3}}\right)\overrightarrow{r}}$$

where

4 $${a}_{p,q,r}=\frac{1}{V}\iiint n\left(\overrightarrow{r}\right){e}^{j\left(p\overrightarrow{{T}_{1}}\left(\overrightarrow{r}\right)+q \overrightarrow{{T}_{2}}\left(\overrightarrow{r}\right)+r\overrightarrow{{T}_{3}}\left(\overrightarrow{r}\right)\right)\overrightarrow{r}}dV$$

The Fourier coefficients ${a}_{p,q,r}$ are complex numbers that quantify the amplitude and offset of each of the planar grating components^3,6^, *V* is the volume of the unit cell, $\overrightarrow{r}$ is the position vector, and *n(*$\overrightarrow{r}$*)* is the refractive index as a function of position. The total range of possible things to spatially vary becomes apparent when the Fourier series parameters are also made functions of the vector position, so Eq. (3) can be written in a vector form as:

5 $$n\left(\overrightarrow{r}\right)={\sum }_{p,q,r}{a}_{p,q,r}\left(\overrightarrow{r}\right){e}^{j\left(p\overrightarrow{{T}_{1}}+q\overrightarrow{{T}_{2}}+r\overrightarrow{{T}_{3}}\right)\bullet \overrightarrow{r}}$$

**Figure 2:**
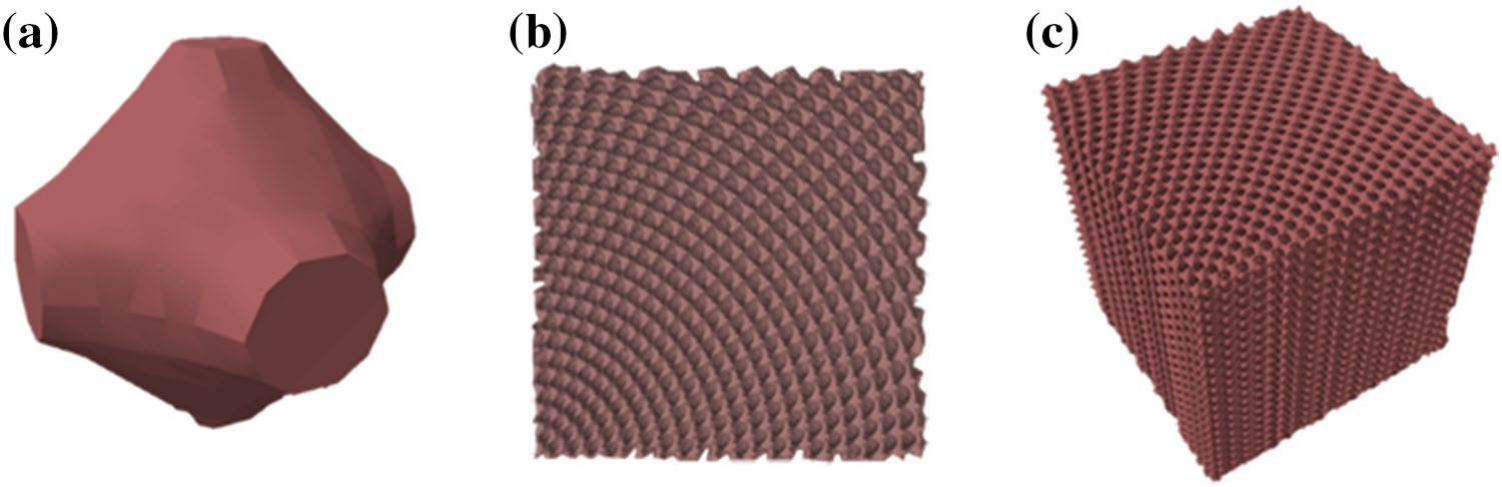
Models for buildup of PC structures. Models were generated for (**a**) Unit cell, (**b**) 2D slab, and (**c**) 3D assembly of SVPC structures.

Using Eq. (5), it is possible to spatially vary all of the attributes, e.g., lattice spacing, orientation of the unit cells, and fill factors, while still generating an overall lattice that is smooth, continuous, and defect free and that minimizes unintentional deformations to the unit cells^4,8^. Once these 2D models are generated, they are loaded into three-dimensional (3D) FDTD for simulation. In order to make 3D PC structures, the 2D slabs can be assembled and stacked on top of one another. The thickness of these slabs is set to be one unit cell in height while being fixed to a negligible value of approximately 1e-7 m for current 2D simulations. The buildup from a single unit cell of a PC to 2D and 3D models is represented in Fig. 2.

The number of layers in these bio-inspired SVPC models was determined by an optimization process to develop a model that has enough layers of holes to maintain self-collimation by creating a structure with a size far greater than the wavelength while not being so large as to make the simulation process inefficient. This optimization process will also be beneficial when moving towards fabrication in the future. The spacing between individual layers is determined by the unit cell size, so the spacing of the holes remain consistent in both the horizontal and vertical directions prior to distortion due to spatial variation. A single unit cell is shown in Fig. 2a, a 2D slab is shown in Fig. 2b, and a full 3D model is shown in Fig. 2c.

These SVPC models can be used for each structure for simulation. The unit cell can be tested for self-collimation properties such as Iso-Frequency Contours (IFCs) discussed later in this paper. 2D slabs and 3D assemblies can be simulated in order to approximate how the structure will function in specific applications such as optical communication systems, LIDAR, and data transfer systems.

In this paper, we outline our approach for the simulation of 2D PC structures. The initial models shown in Fig. 2 are the same models used in the simulations. Unit cells were developed based on material properties and desired fill fractions. Each row of the 2D slab consists of 21-unit cells. Each 2D unit cell has a width and height parameter of 1.5 μm and 3 μm, respectively. These cells are spatially varied based on the desired bend angle to develop the SVPC with a fill fraction of 0.34 while maintaining a smooth curve and continuity between cells to control light.

### Refractive index investigation

In order to control light in the near-IR region, low-refractive index materials such as Silicon Dioxide (SiO_2_) and the polymer SU-8 are ideal for the desired applications, with SU-8 providing a route towards fabrication via 3D lithography. These materials were researched to find the refractive indices and extinction coefficients. As a negative epoxy-based photoresist, SU-8 is an ideal material for lithography fabrication. Its refractive index can be tailored to a range of different wavelengths based on the speed with which it is made and the thickness of the material^9^. Both SiO_2_ and SU-8 have a constant extinction coefficient $\left(k\right)$ of zero. This constant *k* value of zero means that there is no absorption, and both materials will act as loss-less material (consideration of lossy material would require a rigorous treatment that takes into account loss in the bandgap calculation). Since the extinction coefficient was not a factor for our applications in the near-IR range, we focused on the refractive index. SiO_2_ has an approximately constant value of 1.45, and the polymer SU-8 has a constant value of around 1.56 in our area of interest. These values were used in the simulation to determine which material was best suited for our two SVPC models.

If other materials with higher *k* values were used, then energy loss could impact the success of these SVPC structures. This means that greater focus may have to be placed on optimizing the number of layers and unit cells as one would need to find the optimal number of layers to maintain self-collimation and high transmission through bends while also not creating too large of a structure that experiences high loss. Another factor that could impact these parameters is temperature. While temperature variation was not a focus of this work, given the high stability of SU-8 against temperature, temperature has a direct effect on the density of liquid which impacts its refractive index as light travels faster through lower-density materials. Increasing temperature results in a decrease in refractive index, while decreasing temperature results in an increase in refractive index. Additionally, temperature can distort the unit cell size nonlinearly, which can impact the effective index and collimation properties of the SVPC. This means that extreme variations in temperature could have an impact on the effectiveness of the SVPC^10^, although that effect remains a minor one. We will examine the temperature effect while this device is being developed during fabrication. This temperature effect will be reported in future publication.

Varying the material of a PC structure can create a gradient index PC that can enable the control and direction of light in particular wavelengths. Prior work has been done to vary hole thicknesses throughout a structure in order to change the refractive index while one moves through the structure^11^. This variation in hole thickness is similar to the work performed in this paper. As unit cells are spatially varied throughout our model, the shape and size of holes are stretched and changed as one moves through the structure. This manipulation of holes has a direct impact on the effective index in that position of the structure in a similar way to gradient index PCs^11^.

Other optical devices such as mirrors, waveguides, or optical fibers can also be used to direct specific wavelengths of light. However, these devices can introduce high losses that are not present in the SVPCs designed throughout this work. Additionally, these devices do not have the same self-collimation properties developed by these SVPC structures that can enable the desired applications.

### Anti-reflection coatings

The difference in the refractive index between the material of interest and the environment, e.g., air, can cause high reflection at the interface of the PC. In order to reduce losses due to reflection, Anti-Reflection Coatings (ARCs) were introduced to help ease the light into the structure. ARCs are made up of one or more thin layers placed on the interface of the structure^12^ with a refractive index found using Eq. (6):

6 $${n}_{ARC}=\sqrt{{n}_{1}{n}_{2}}$$

The width, or thickness, of the ARC is related to the wavelength of interest ($\lambda$) of the applied incident beam as shown in Eq. (7):

7 $$d=\frac{1}{4}\lambda$$

Using Eqs. (6) and (7), we can generate an ARC with a refractive index between the two materials that would allow the beam to be eased into the structure and reduce the amount of loss from reflection at the input interface of the SVPC in 1D, 2D, and 3D PCs^13^. For practical applications and fabrication where a single structure may be used for multiple wavelengths, the thickness can be selected for an average wavelength or the most common wavelength used in the system.

A few different geometries were considered for these ARCs in various applications. The simplest form, a rectangular structure, was used for logic gate applications that could allow multiple positions or incident angles to be used. In order to optimize the transmission for the 90-degree bend, a tapered cone was used as the ARC structure to give a specific point of entry to guide the beam into the SVPC structure. Both of these ARCs were able to reduce the amount of reflection at the input and help guide various wavelengths of light into the respective structures and improve the transmission results. While more complex waveguides such as the tapered cone used for the 90-degree bend provide the greatest improvement in transmission, their complexity in both fabrication and implementation may limit its potential applications.

### Numerical modeling with the 2D FDTD method

To study the self-collimation and transmission of multiple SVPC structures for different applications, the FDTD method can be used in commercial software such as Lumerical. 2D structures were developed through a combination of MATLAB and CAD software in SolidWorks and Lumerical. MATLAB was first used to develop basic geometries prior to being imported into SolidWorks, so individual layers and cells could be stacked, simplified, or scaled to fit specific parameter requirements for Lumerical FDTD. Using unit cells of these lattice structures, we could develop a model to observe its bandgaps and predict its ability to maintain self-collimation of different wavelengths of light. These results helped us to create simulation models to find the transmission and reflection characteristics for different wavelengths of each application. Starting with 2D simulations is reasonable as 3D simulations will be too time-intensive for preliminary analysis.

## Results and discussion

### PC bandgap analysis

**Figure 3:**
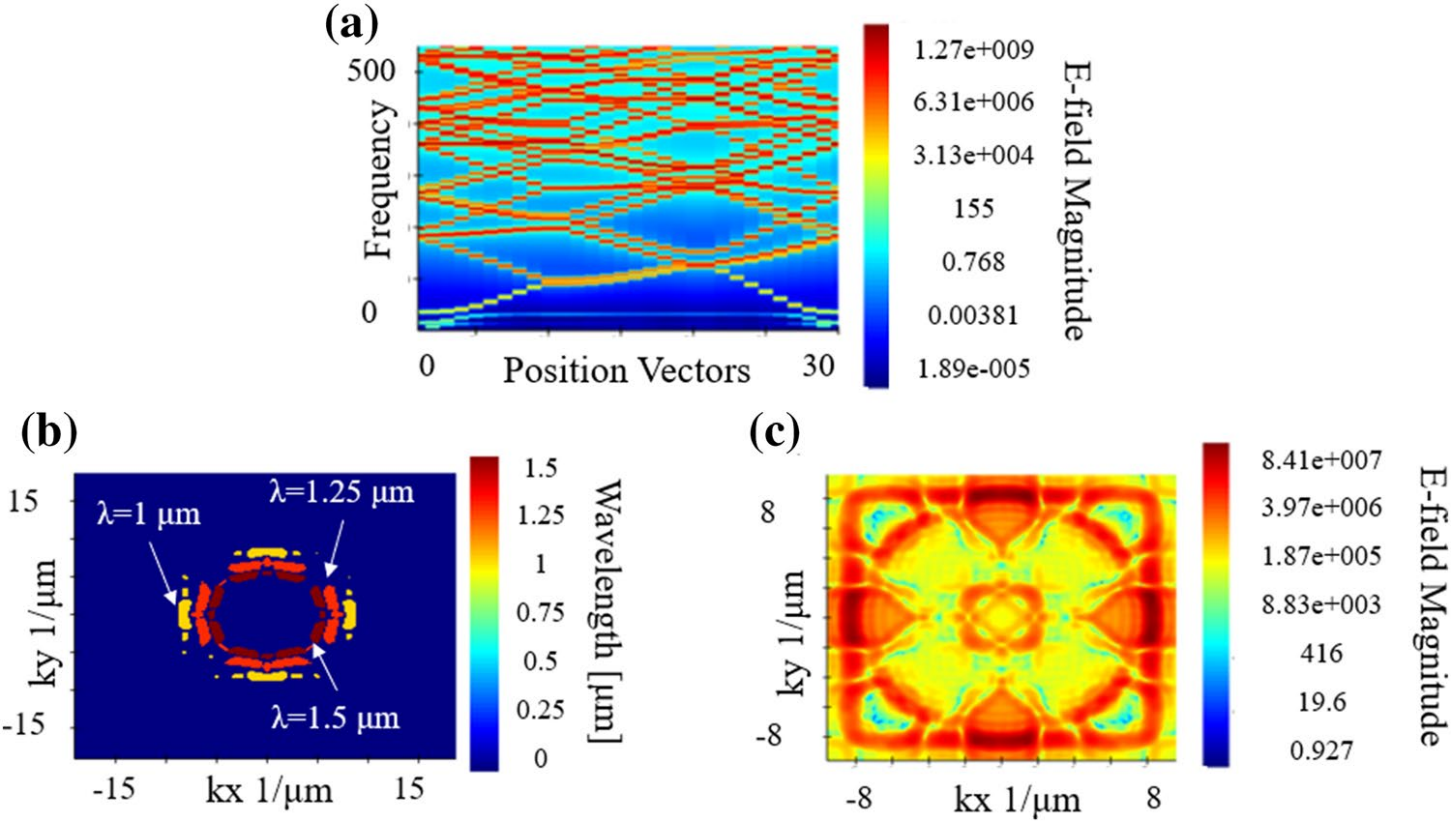
FDTD models of SiO_2_ PC unit cell for (**a**) band structure, (**b**) IFC for multiple wavelengths, and (**c**) IFC at 1000 nm.

To show the ability of these bio-inspired SVPCs in applications for beam steering and logic gates, we can take advantage of bandgaps and self-collimation of the PC structures. PCs can affect the motion of photons, so we can tune the light in a desired direction through a 90-degree bend. The ability to distort or bend light is dependent on the photonic bandgap of the structure and the density of $\overrightarrow{K}$-vectors as a function of angular directions. The photonic bandgap is a range of frequencies in which certain wavelengths of light are blocked by a structure^14^. Photons of wavelengths lying in the photonic bandgap cannot propagate through the structure because they do not match the frequency needed to propagate. The bandgap for SiO_2_ is shown in Fig. 3a. This bandgap shows which range of frequencies will be able to propagate through the designed SVPC structure and which will be restricted by its structural and optical properties. Taking advantage of this property, the flow of light can be controlled by prohibiting or allowing light to flow in certain directions in $\overrightarrow{K}$-space. This flow of light is specifically controlled through tailoring the geometric structure and material of the SVPC to manipulate the bandgap. The ability of the SVPC to force light in specific directions as a result of the density of $\overrightarrow{K}$-vectors enables self-collimation throughout the structure^14^. Moreover, by adiabatically introducing a rotation to the PC, we can manipulate the direction of the flow of light without leaving the autocollimation regime. This innovation will make it possible to replace waveguides in the future by this type of self-guiding.

The capability of self-collimation can be shown through IFCs; these contours provide insight into understanding key optical concepts such as negative refraction, super collimation, and super lensing. The angular distribution of the scattered photons leads to the formation of IFCs in the far field. Light passes through perpendicular to the contours of the IFC. The distribution of $\overrightarrow{K}$-vectors in the images of Fig. 3b,c show that as long as the light enters within that range, $\overrightarrow{K}$-vectors will force light to travel in the same direction. By limiting the input angle, we can ensure that the light travels along the same path and remains collimated throughout the entire structure. Similarly, by limiting the spatial variation between each unit cell, we can maintain this self-collimation throughout the full SVPC structure. The density of these $\overrightarrow{K}$-vectors relates to flat bands in IFCs that show areas of the cell that are nondispersive^15^, meaning that these flat portions of the IFC will permit light to remain collimated as it passes through this surface of the unit cell. Prior work has shown the promise of IFCs to show self-collimation at wavelengths in the microwave region. In this work, we expanded this application to the near-IR range and showed that IFCs can maintain self-collimation through our SVPC designs. Using 3D FDTD, we can focus on a single unit cell of our structure to find the bandgap and IFCs for SiO_2_ as shown in Fig. 3.

**Figure 4:**
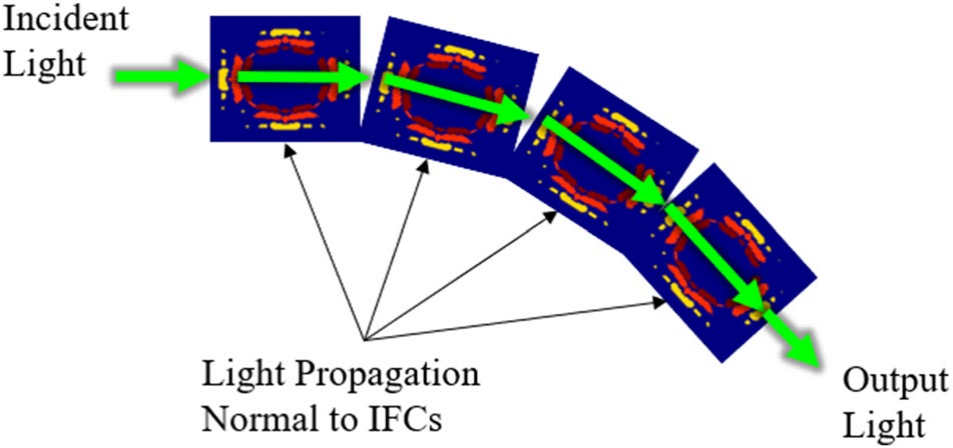
Light propagation through unit cell IFCs.

The results shown in Fig. 3 allow us to predict how the PC will respond when subjected to a certain frequency. The vectors $\overrightarrow{{k}_{x}}$ and $\overrightarrow{{k}_{y}}$ denote the *x* and *y* components of the $\overrightarrow{K}$-vector. In order to maintain self-collimation through our unit cell, the beam divergence angle must be kept below ± 14.03 degrees. The beam must have a constant value of -8 [1/μm] for $\overrightarrow{{k}_{x}}$, while $\overrightarrow{{k}_{y}}$ can range between -2 and 2 [1/μm] because in this region the density of the $\overrightarrow{K}$-vectors is the strongest. As long as these parameters are followed, self-collimation should be maintained throughout the structure. Experimental work has been done on similar PCs that can be used to verify the trends for these bandgaps and IFCs for our structures^16^. Through spatial variation of these unit cells while staying within these $\overrightarrow{K}$-vector ranges, light can be directed through bends as shown in Fig. 4.

The $\overrightarrow{K}$-vector range presented by these IFCs also has a direct impact on the angle of incidence. From these results, we see that the angle of perfect incidence and transmission would be the angle normal to the surface of the unit cell and overall SVPC structure. The $\overrightarrow{{k}_{x}}$ and $\overrightarrow{{k}_{y}}$ vectors show that there should be a range of incidence angles that have high transmission, meaning that this should be a future area of study to look at the influence of incidence angle on the transmission and reflection properties of this SVPC. We can take advantage of these properties and design characteristics to develop SVPCs for specific applications in beam steering.

### The 90-degree bend

**Figure 5:**
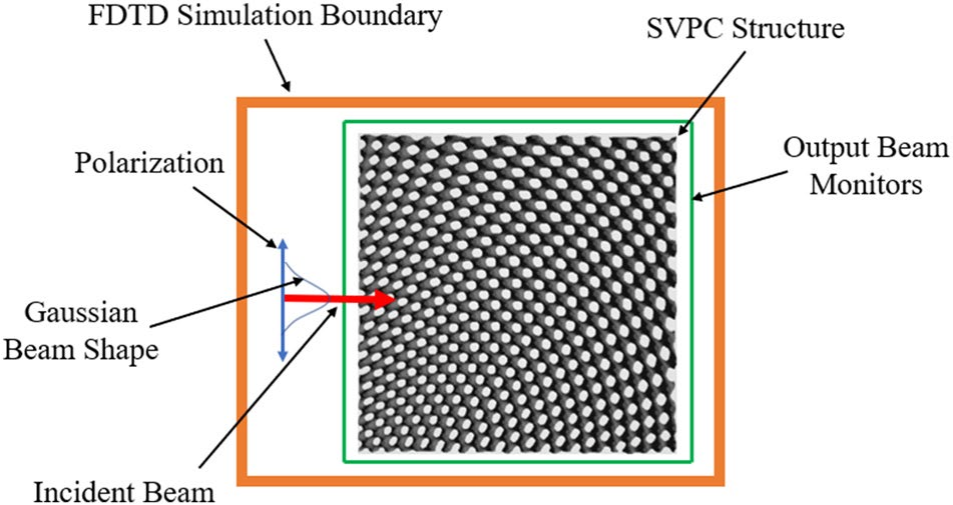
2D SVL simulation: Blue arrows indicate the polarization of the beam; the red arrow indicates the propagation direction of the input beam. The orange and green borders show the FDTD simulation boundary and monitors for transmission and reflection, respectively.

Our first SVPC model was designed with a 90-degree bend for beam-steering applications. Generally, bending light in an integrated structure requires fabricating waveguides specific to each wavelength, meaning multiple input beams would require multiple waveguides. Through self-collimation, it is possible to bend light without necessitating a waveguide geometry, significantly reducing the complexity of fabrication. Our design was modeled to maintain self-collimation of light in the near-IR range through a 90-degree bend. This model, shown in Fig. 1a, was loaded into FDTD for simulation. Parameters were set to test a range of wavelengths from 600 to 1000 nm with monitors set at each face to find the absorption, reflection, and transmission through the different faces. This 2D model is shown in Fig. 5.

Using this model, we were able to test two different materials: SiO_2_ and SU-8. This testing gave a range of results that could fit different fabrication methods. Materials such as SiO_2_ can be fabricated by etching into material substrates, while the polymer SU-8 could be used for more exact lithography and printing.

#### Numerical modeling and simulation

**Figure 6:**
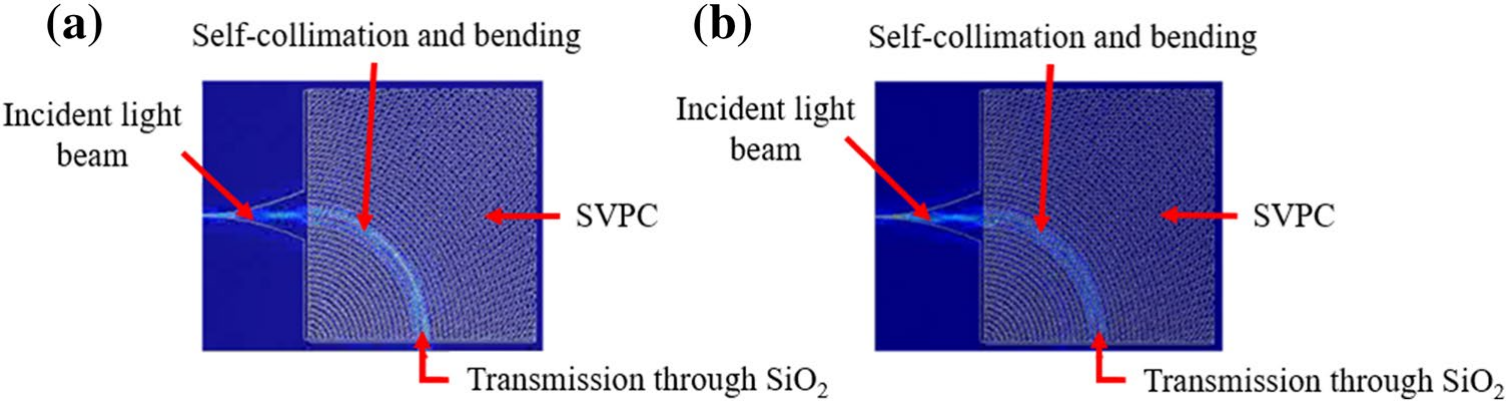
Simulation model for light passing through SVPC structure. Similar results were found for both (**a**) SiO_2_ and (**b**) SU-8.

With this information, we can generate the bio-inspired SVPC structures and perform FDTD simulations in Lumerical. A structure was generated and tested with each material from 800 to 1000 nm. Figure 6 shows that the SVPC structure for (a) SiO_2_ and (b) SU-8 was able to keep the incident light self-collimated as it bent 90 degrees through the structure.

**Figure 7:**
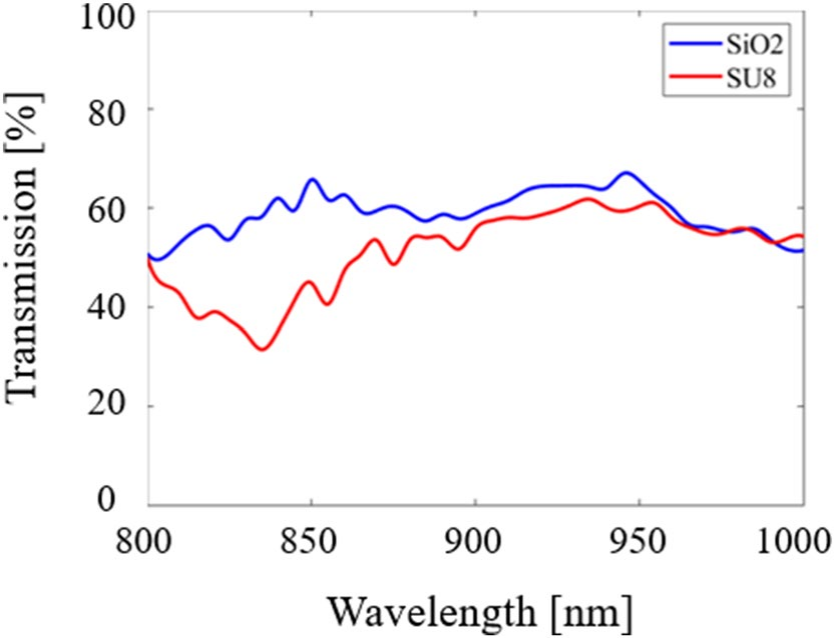
Transmission results (in percentage) for SVPCs designed with SiO_2_ and SU-8 in the near-IR region from 800 to 1000 nm using FDTD simulations in Lumerical.

The light is shown to be slightly brighter for the SiO_2_ model as it was able to achieve higher transmission due to a lower refractive index. Transmission and reflection data were calculated every 10 nm in each structure. The results for transmission are shown in Fig. 7.

This data allows us to better understand how our SVPCs may operate and which materials are best suited for beam-steering applications such as optical communication and multiplexing and demultiplexing. These results show that SiO_2_ is able to achieve significantly higher transmission than SU-8; this is a result of the difference in refractive index as SiO_2_ has a much lower value of approximately 1.45, while SU-8 is higher at 1.56. The peak values for each material are presented in Table 1; please note that the summation of all transmission and reflection data is not exactly equal to 100% due to rounding the values for each set of transmission to two decimals.

**Table 1:** Peak transmission results (in percentage) for SVPCs designed to control light through a 90-degree bend with SiO_2_ and SU-8.

Material	Peak Wavelength [nm]	Bent Transmission [%]	Transmission through SVPC [%]	Transmission Deflected Upward [%]	Reflection [%]
SiO_2_	945	66.99	10.00	12.71	10.31
SU-8	935	61.76	12.28	11.07	14.88

Using these results, we can select which material is best suited for operation in specific wavelengths and understand how we can tune the PC to operate under different conditions.

#### Comparison with published results

**Figure 8:**
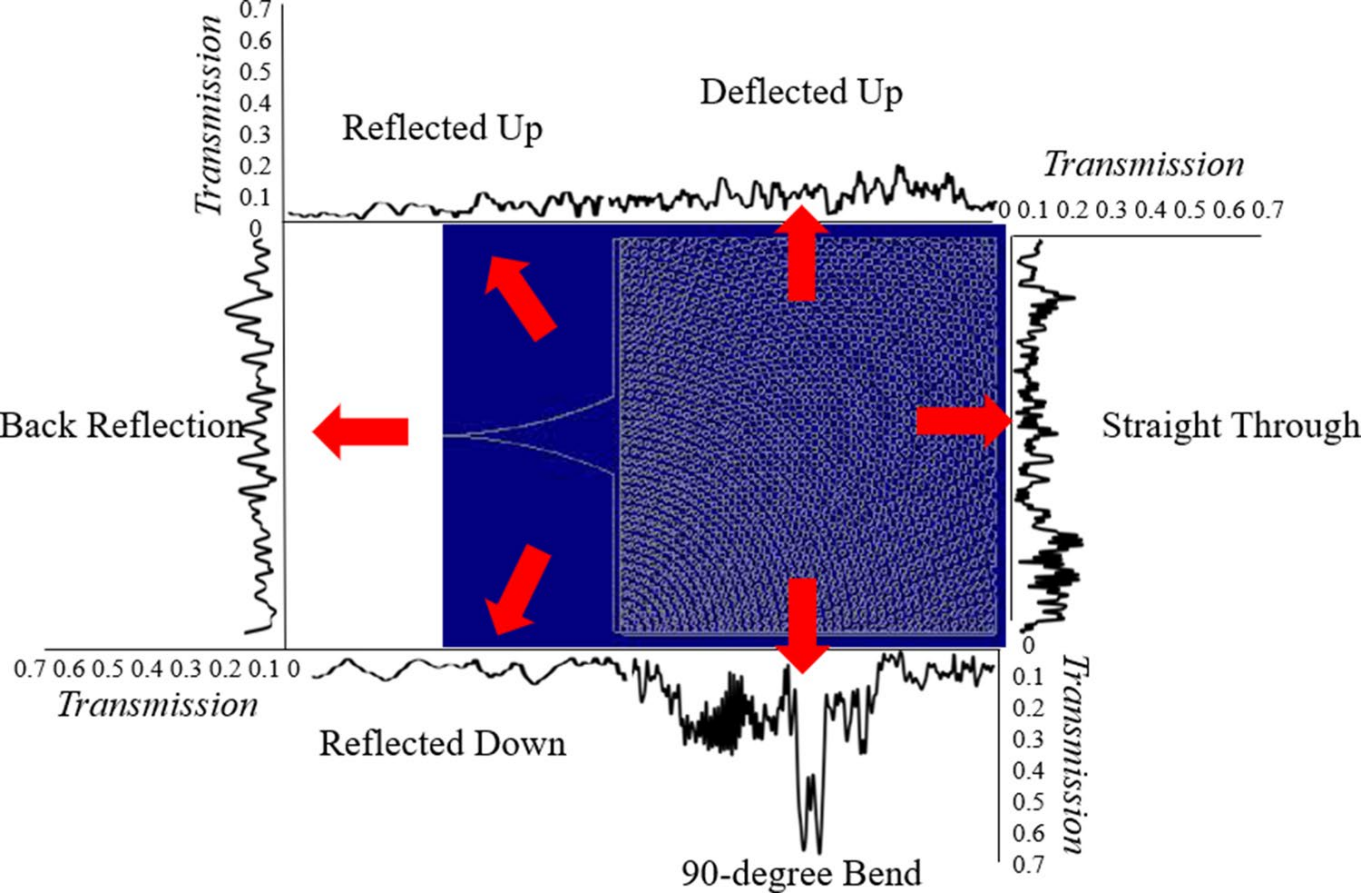
Comparison between transmission through each face of the SVPC structure.

As mentioned above, similar SVPC designs have been developed in the past to operate in the visible and microwave spectrums. While we cannot compare these results with other SVPCs in our targeted spectrum, we can contrast the trends between the results of our models with those published for other wavelengths^1,7^. Figure 8 shows the comparison of transmitted light through different faces of our model’s structure.

These results show that our work has been able to follow similar trends as those found in published work^1,7^ and has improved the results of controlled transmission through the bend. While SVPCs operating at wavelengths around 2.94 μm were shown to be capable of transmitting a maximum E-field magnitude of nearly 0.25, we were able to increase the number of unit cells and utilized ARCs to raise this value to a magnitude of around 0.65 in the near-IR spectrum. Similar trends for decreased reflection and transmission straight through the structure were also seen. Performing fabrication and testing at our desired wavelengths will allow this comparison to be fully verified with those from other published works.

### Optical logic gate

**Figure 9:**
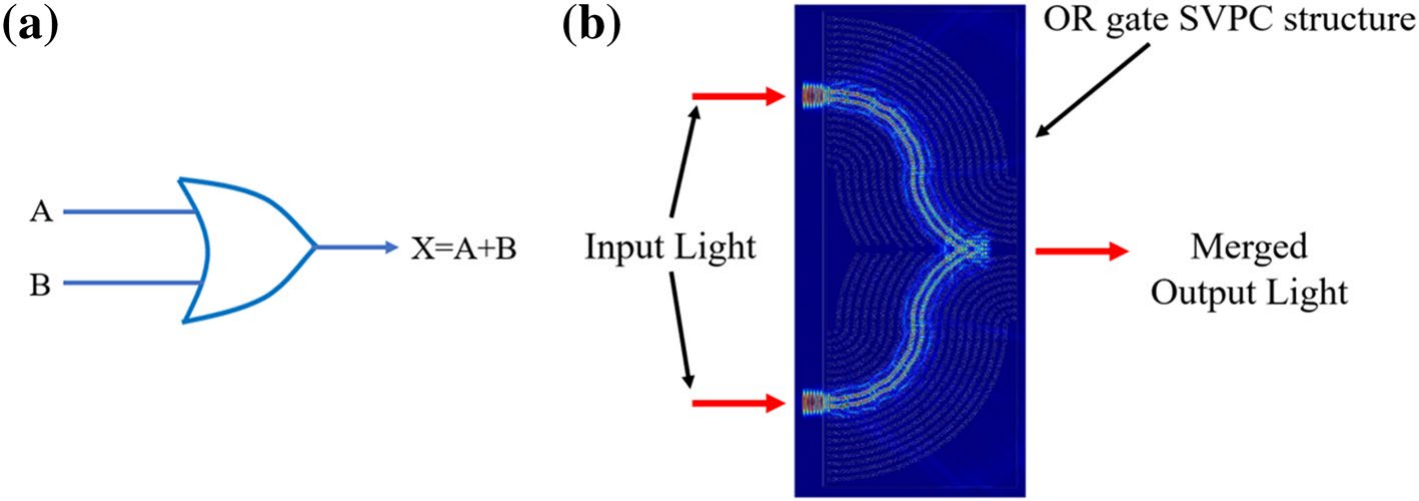
(**a**) Electronic OR gate; (**b**) 2D SVPC model for optical OR gates.

The second SVPC model was designed for optical logic gates based on linear optics, specifically OR/AND gates. The same principle of self-collimation allows the SVPC to direct two separate light beams along the same path in order to function as an OR gate (Fig. 9); this can be done using two input beams of either the same or different wavelengths within the near-IR range. Essentially, when both beams are in the High state (on), the merged output exceeds the threshold for a logic high, while if either of them is in the Low state (off), the output drops below the threshold for a logic high. Implementing an SVPC that can direct light for this application through a geometric structure and a material’s refractive index could allow these optical logic gates to be implemented into optical systems more often and more easily without the need of larger, heavier optical hardware. This model, shown in Fig. 9b, was generated in FDTD using a similar method for spatially varying unit cells for the 90-degree bend. As shown in Fig. 9a, an electronic logic OR gate is compared with an optical OR gate using a PC with nanocavity-enabled logic gates.

This structure should be able to maintain self-collimation as it guides two different beams from multiple inputs to a single output. Utilizing low-refractive index materials and the geometric structure will show the potential for the SVPC to function for applications with optical logic gates.

#### Numerical modeling and simulation

**Figure 10:**
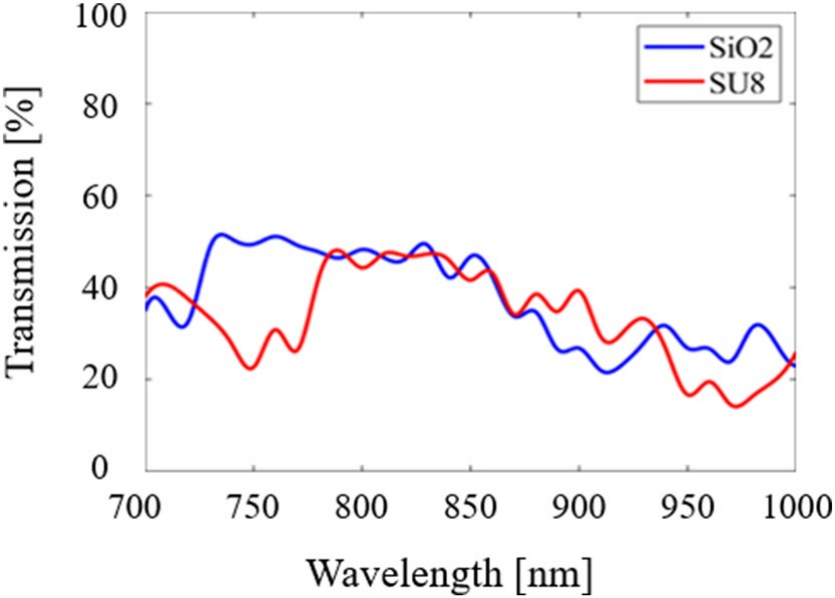
Transmission results for optical logic gate (OR gates) with two different low-refractive index materials. Results for SiO_2_ and SU-8 are shown by the blue and red lines, respectively.

Using the model in Fig. 9b, material properties were assigned and tested from 700 to 1000 nm. Input beams using these wavelengths were sent from two entry points as shown in Fig. 9b and used self-collimation to merge the light into a single output. While some optical logic gates function with input beams of different wavelengths, simulations were conducted where both beams used the same input wavelength. These and gate simulations were conducted using the same materials as our previous simulations: SiO_2_ and SU-8. The results from these simulations are shown in Fig. 10.

A corresponding truth table is given in Table 2 for our logic gate. For example, when the detector threshold at the output is set to 50% of the maximum input, the gate acts as an OR gate. If it is set to 100% of the maximum input in either arm, it acts as an AND gate.

**Table 2:** Truth table for operation at 780 nm with output corresponding to setting the detection threshold at 50% or 100% of the maximum input.

A	B	Threshold at 50%	Threshold at 100%
Low	Low	Low	Low
High	Low	High	Low
Low	High	High	Low
High	High	High	High
Operation		OR	AND

These results show that the highest transmission was achieved using SiO_2_ at 760 nm with 51.1% of the input light collimating and merging into a single output and at 780 nm for SU-8 (~ 50%). The transmission results for logic gates were not as consistent throughout the near-IR range as they were for the simpler 90-degree bend application; this decrease in consistent transmission across the near-IR range is likely due to the multiple bends required to develop the OR gate structure. SiO_2_ was able to achieve the highest overall transmission values and have a consistently higher value across a majority of the near-IR spectrum than SU-8. Similar to SiO_2_, however, the values for SU-8 were also not as consistent as the values generated with our first model. Adjusting the hole size and spacing for the variation in the lattice could lead to more consistent, improved results. This work will be pursued in the future to show the added benefits of SVPCs for optical logic gates.

**Figure 11:**
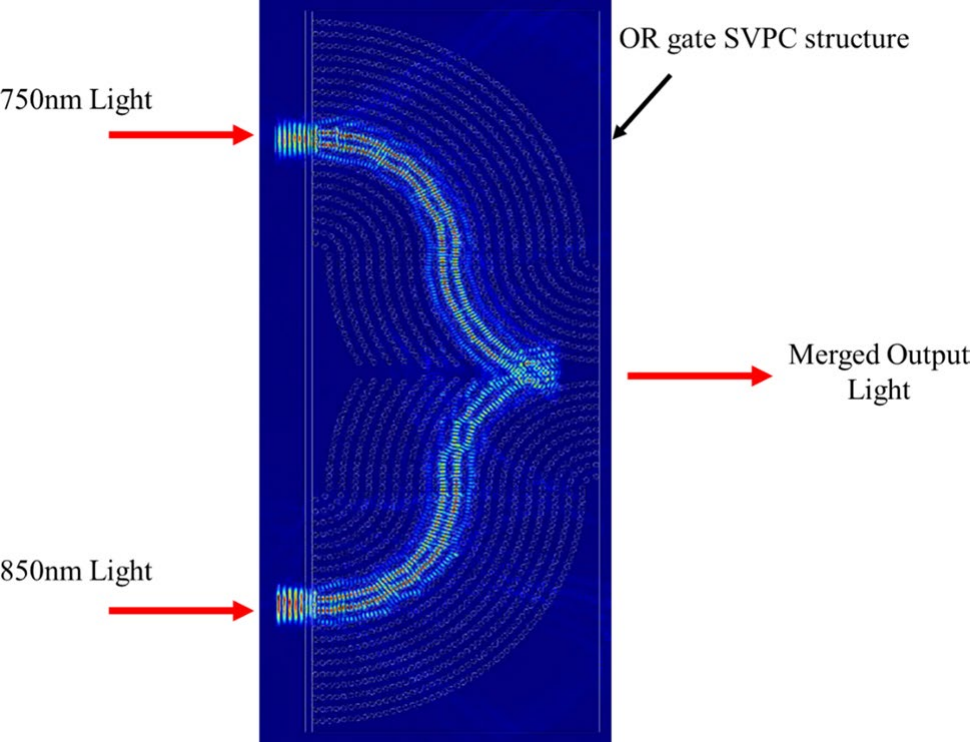
SiO_2_ SVPC optical logic gate for two dissimilar wavelengths.

Another benefit of logic gates is the ability to control multiple dissimilar wavelengths at the same time. To show this ability, a simulation was run with one beam of incident light set to 750 nm and the second set to 850 nm as shown in Fig. 11. The SVPC’s ability to multiplex multiple inputs of dissimilar wavelengths into a single output makes it ideal for logic gate applications. This SVPC-based optical logic gate has advantages over waveguide-based logic gates in that it can be designed to fit a wider range of wavelengths and can utilize multiplexing and demultiplexing to operate with multiple inputs.

This result shows that even with two dissimilar wavelengths, a single SVPC structure can be used to maintain self-collimation while directing and merging light together. The transmission and reflection values are shown in Table 3.

**Table 3:** Transmission and reflection results for SVPC optical logic gate with dissimilar wavelengths.

Material	Wavelength [nm]	Bent Transmission [%]	Transmission through SVPC [%]	Reflection [%]
SiO_2_	750 and 850	50.31	17.31	32.39
SU-8	750 and 850	35.33	16.08	48.58

These results for dissimilar wavelengths show the potential benefits of this SVPC design. SiO_2_ was significantly more successful than SU-8 with 50.31% transmission compared to 35.33%. This difference is likely due to the consistently higher results of SiO_2_. Though SU-8 was able to achieve high results with similar wavelengths, these values were not as consistent as those for SiO_2_. This means that SiO_2_ could have a greater benefit for logic gate applications using dissimilar wavelengths such as multiplexing and demultiplexing but at the expense of more complex fabrication as the material does not readily lend itself to 3D lithography.

## Discussion

This work has shown the ability to design bio-inspired SVPCs for specific optical applications in the near-IR range which is novel; these devices have only previously been operated in the visible and microwave wavelengths. Using low-refractive index materials such as SiO_2_ or SU-8, SVPCs can be designed based on SVLs found in butterfly wings and fish scales in order to maintain self-collimation of light through sharp bends; this can be shown through slow variation and bends between individual unit cells and their respective IFCs in order to keep high transmission through these bends. These capabilities show promise for SVPCs in applications such as beam steering and logic gates. These SVPCs could be integrated into optical communication systems, data transfer, or multiplexing and demultiplexing. Through their geometric design and small weight, these structures continue to show their advantage over standard optical equipment and waveguides for the previously described applications. In the current work, initial models for these applications are being fabricated to verify our results with the experimental results and to begin moving towards implementation of SVPCs for new optical systems. The experimental results are planned to be reported in peer reviewed journals in the near future.
